# Functional gastrointestinal disorders in adults with epilepsy: associations with psychological symptoms, chronotype, and seizure burden

**DOI:** 10.1007/s11845-026-04396-x

**Published:** 2026-05-06

**Authors:** Gizem Nur Solak Khan, Ferda Ilgen Uslu

**Affiliations:** 1 Faculty of Health Sciences Department of Neuroscience, Bezmialem Vakıf University, İstanbul, Türkiye; 2 Istanbul Faculty of Medicine, Department of Neurology, Division of Clinical Neurophysiology, Istanbul University, Istanbul, Türkiye; 3 İstanbul Üniversitesi, İstanbul Tıp Fakültesi, Yeni Nörolojik Bilimler Binası, Fatih, İstanbul Turkey

**Keywords:** Epilepsy, Functional gastrointestinal disorders, Irritable bowel syndrome, Functional constipation, Anxiety and depression, Chronotype

## Abstract

**Background:**

Epilepsy is frequently accompanied by psychiatric comorbidities, whereas functional gastrointestinal disorders are often overlooked. This study investigated the prevalence of functional gastrointestinal disorders in adult patients with epilepsy (PWE) and their associations with psychological symptoms, chronotype, and seizure characteristics.

**Methods:**

In this cross-sectional case–control study, 100 adult PWE and 100 age- and sex-matched healthy controls (HC) were evaluated. Functional gastrointestinal disorders were assessed using Rome IV criteria and stool form with the Bristol Stool Form Scale. Depression and anxiety were measured with the Beck inventories, and chronotype with the Morningness–Eveningness Questionnaire. Clinical epilepsy variables and antiseizure medication profiles were recorded.

**Results:**

Functional gastrointestinal disorders were significantly more prevalent in PWE than in HCs. Irritable bowel syndrome and functional constipation occurred more frequently in the PWE, and constipation-predominant stool patterns were markedly increased. Patients with epilepsy showed higher rates of moderate-to-severe depression and anxiety and a predominance of intermediate chronotype. Within the PWE, functional gastrointestinal disorders and constipation-predominant stool patterns were associated with higher psychological symptom burden, polytherapy, and ongoing seizures, but not with epilepsy subtype, electroencephalography, or magnetic resonance imaging findings.

**Conclusion:**

Adults with epilepsy exhibit a substantially increased burden of functional gastrointestinal disorders accompanied by elevated psychological symptoms and altered chronotype distribution. Gastrointestinal symptoms were largely unreported clinically, despite their association with greater disease complexity. Systematic screening for functional gastrointestinal disorders, mood symptoms, and chronotype may facilitate a more comprehensive and integrated approach to epilepsy care. These findings underscore the need for multidisciplinary assessment in practice.

## Introduction

Epilepsy is a chronic neurological disorder characterized not only by recurrent seizures but also by a broad range of psychiatric and somatic comorbidities that substantially impair quality of life. Among these, depression and anxiety are the most consistently recognized and clinically addressed comorbid conditions, with prevalence rates exceeding those observed in the general population [1, 2]. In contrast, gastrointestinal complaints are often overlooked in routine epilepsy care, despite their potential impact on daily functioning and overall disease burden.

Functional gastrointestinal disorders (FGIDs), including irritable bowel syndrome (IBS) and functional constipation, are common conditions in the general population and are strongly associated with psychological distress through bidirectional gut–brain interactions [3]. IBS, in particular, has been linked to altered autonomic regulation, heightened visceral sensitivity, and dysregulation of central stress pathways. These mechanisms overlap with neurobiological processes implicated in epilepsy, suggesting that FGIDs may represent a relevant but underrecognized comorbidity in patients with epilepsy.

Emerging evidence indicates that adults with epilepsy experience FGIDs more frequently than individuals without epilepsy. Clinical studies have demonstrated higher rates of IBS and constipation in epilepsy cohorts, with some reporting associations between gastrointestinal symptoms and seizure frequency or disease severity [4]. Moreover, population-based data suggest that IBS may precede the development of epilepsy, supporting the hypothesis of shared pathophysiological pathways rather than a purely secondary phenomenon [5]. The gut–brain axis, encompassing neural, immune, endocrine, and microbiota-related signaling, has been proposed as a central framework linking gastrointestinal dysfunction with altered neuronal excitability and seizure susceptibility [6].

Sleep and circadian rhythm disturbances constitute another important but relatively underexplored dimension of epilepsy. Seizures often display circadian patterns, and disrupted sleep–wake regulation has been associated with increased seizure frequency and impaired mood regulation [7]. Chronotype, reflecting individual circadian preference, has been linked to emotional well-being and behavioral regulation in the general population, with intermediate and evening chronotypes associated with higher levels of anxiety and depressive symptoms [8]. However, data examining chronotype distribution in adult epilepsy populations, particularly in relation to gastrointestinal symptoms and psychological burden, remain limited. Circadian rhythm disruptions may influence gastrointestinal motility, intestinal permeability, and microbiota composition, thereby providing a plausible mechanistic link between chronotype, gastrointestinal symptoms, and neuropsychiatric regulation [9]. Although the associations between epilepsy and psychiatric comorbidities, gastrointestinal disorders, and sleep disturbances have been investigated separately, studies integrating these domains within the same adult epilepsy cohort are scarce. A more comprehensive evaluation of these interrelated factors may provide insight into the multidimensional burden of epilepsy and help identify clinically relevant, yet often neglected, comorbidities.

In addition, several antiseizure medications are known to have gastrointestinal side effects, including constipation, nausea, and dyspepsia, which may further contribute to the burden of gastrointestinal symptoms in patients with epilepsy [10].

Therefore, the present study aimed to evaluate functional gastrointestinal symptoms in adults with epilepsy and to examine their associations with psychological comorbidities, chronotype, seizure characteristics, and antiseizure treatment profiles. Using validated clinical instruments and an age- and sex-matched healthy control group, this study seeks to provide an integrated assessment of gastrointestinal, psychological, and chronobiological features in epilepsy.

## Methods

### Study design and participants

This cross-sectional, case–control study was conducted at the Epilepsy Outpatient Clinic of Bezmialem Vakıf University, Department of Neurology, between October 2022 and February 2023.

The pre-test sample size calculation was performed using GPower version 3.1. Since a difference of approximately 25% in the prevalence of functional gastrointestinal disorders was expected between epilepsy patients and the healthy control group, a total sample size of at least 126 participants (63 in each group). To account for potential exclusions and increase the robustness of subgroup analyses, a final sample size of 100 epilepsy patients and 100 healthy controls was included.

Consecutive adult patients (≥ 18 years) with a confirmed diagnosis of epilepsy according to International League Against Epilepsy (ILAE) criteria and a minimum follow-up duration of one year were enrolled. A control group of healthy individuals matched for age and sex, with no history of epilepsy or major neurological disorders, was recruited. Patients with suspected seizures, first seizures, acute symptomatic seizures, syncope, psychogenic nonepileptic seizures, severe cognitive impairment, active psychotic disorders, known organic gastrointestinal diseases, inflammatory bowel disease, or systemic illnesses that could significantly affect gastrointestinal function were excluded. Participants were also screened for the use of medications that could affect gastrointestinal motility, including proton pump inhibitors, laxatives, and antidepressants, and this information was considered in the interpretation of the findings. All participants provided written informed consent. The study was approved by the local ethics committee.

### Clinical data collection

Demographic characteristics were recorded for all participants. For patients with epilepsy, clinical data including seizure type (focal, generalized, or unclassified), epilepsy syndrome classification (Focal, genetic generalized, unknown), seizure frequency within the previous year, disease duration, antiseizure medication (ASM) use, number of ASMs, and ASM profile were obtained through medical records and patient interviews. ASMs were categorized as sodium channel blockers (SCB) or non–SCBs according to pharmacological properties. This classification was used to explore potential differences in gut–brain interactions, as sodium channel modulation may influence autonomic and neuronal excitability pathways relevant to gastrointestinal function. Electroencephalography (EEG) and brain magnetic resonance imaging (MRI) results were classified as normal or pathological.

### Gastrointestinal assessment

Functional gastrointestinal symptoms were assessed using the Rome IV diagnostic criteria. Participants were classified as having irritable bowel syndrome (IBS), functional constipation (FC), or no functional gastrointestinal disorder [11]. Stool form was evaluated using the Bristol Stool Form Scale, and constipation-predominant stool patterns were defined as Bristol stool types 1–2 [12].

### Psychological assessment and chronotype

Psychological status was evaluated using validated Turkish versions of the Beck Depression Inventory (BDI) and Beck Anxiety Inventory (BAI) [13–16]. 

Chronotype was evaluated using the Morningness–Eveningness Questionnaire (MEQ) and categorized as definite morning type, morning-leaning type, intermediate type, evening-leaning type, or definite evening type [17, 18]. For statistical analyses, chronotype categories were recoded into three groups to improve interpretability and address small subgroup sizes: morning types (definite morning and morning-leaning), intermediate type, and evening types (evening-leaning and definite evening).

All questionnaires were administered to patients and controls under similar conditions.

### Statistical analysis

Statistical analyses were performed using IBM SPSS Statistics version 24.0. Categorical variables were summarized as counts and percentages, while continuous variables were expressed as mean ± standard deviation or median (interquartile range), as appropriate. Group comparisons between patients with epilepsy and healthy controls were performed using chi-square or Fisher’s exact tests for categorical variables, with Fisher’s exact test applied when expected cell counts were less than 5, and Student’s t-test or Mann–Whitney U test for continuous variables, depending on data distribution. Within the epilepsy cohort, subgroup analyses were conducted according to Rome IV gastrointestinal status (IBS, functional constipation, and no functional gastrointestinal disorder). Comparisons across these subgroups were performed using chi-square tests and interpreted descriptively due to small subgroup sizes. Exploratory logistic regression analysis was performed to evaluate factors independently associated with IBS, with IBS status entered as the dependent variable. Independent variables included epilepsy status, anxiety severity, depression severity, and chronotype category. Correlation analyses were conducted using Pearson or Spearman correlation coefficients as appropriate. A p value ≤ 0.05 was considered statistically significant. Subgroup analyses were exploratory; therefore, p-values are presented for transparency and interpreted cautiously, particularly given the potential for multiple comparisons.

## Results

The study included 100 adults with epilepsy (PWE) who were followed at a tertiary epilepsy outpatient clinic and 100 age- and sex-matched HCs (HCs). The epilepsy group consisted of 46 males (46%) and 54 females (54%), with a mean age of 34 years (range: 18–69). The control group included 32 males and 68 females, with a mean age of 37 years (range: 18–74). There were no significant differences between groups with respect to age or sex distribution (*p* = 0.212 and *p* = 0.420, respectively).

According to the 2017 International League Against Epilepsy classification, 45 patients (45%) were classified as having focal epilepsy, 28 (28%) generalized epilepsy, and 27 (27%) unclassified epilepsy. Among patients with focal epilepsy, temporal lobe epilepsy was identified in 21 individuals. During the preceding year, 55 patients (55%) had remained seizure-free, whereas 45 (45%) had experienced at least one seizure. Regarding antiseizure medication (ASM) use, 66 patients (66%) were receiving monotherapy, while 33 patients were treated with polytherapy. Levetiracetam was the most frequently used ASM, followed by carbamazepine/oxcarbazepine, lamotrigine, and valproate. Sodium channel–blocking ASMs were used by 53 patients, whereas 43 patients received other ASMs; 4 patients were treated with both classes.

Based on the Beck Depression Inventory, moderate-to-severe depressive symptoms were significantly more prevalent in PWE compared with HCs (53% vs. 4%, *p* < 0.001). In the epilepsy group, moderate-to-severe depression was more common in females than in males (64.8% vs. 39.1%, *p* = 0.010). Depression severity did not differ significantly according to seizure type, seizure frequency, the number of ASM, classification of ASM, EEG findings, or MRI findings (all *p* > 0.05) (Table 1).

**Table 1:** Clinical, psychological, gastrointestinal, and chronobiological characteristics of epilepsy patients and controls

Variable	Epilepsy (*n* = 100)	Controls (*n* = 100)	*p* value
Sex, n (%)			0.420
Female	54 (54)	68 (68)	
Male	46 (46)	32 (32)	
Age, years	34 (18–69)	37 (18–74)	0.212
Epilepsy type, n (%)			—
Focal epilepsy	45 (45)	—	
Generalized epilepsy	28 (28)	—	
Unclassified epilepsy	27 (27)	—	
Seizure-free ≥ 1 year, n (%)	55 (55)	—	
ASM therapy, n (%)			
Monotherapy	66 (66)	—	
Polytherapy	33 (33)	—	
Moderate–severe depression, n (%)	53 (53)	4 (4)	< 0.001
Moderate–severe anxiety, n (%)	54 (54)	18 (18)	< 0.001
Bristol stool type, n (%)			
Constipation-predominant (Types 1–2)	27 (27)	5 (5)	< 0.001
Rome IV diagnosis, n (%)			
No FGID	59 (59)	86 (86)	
Irritable bowel syndrome	14 (14)	6 (6)	< 0.001
Functional constipation	27 (27)	8 (8)	< 0.001
Chronotype, n (%)			< 0.001
Morning type	10 (10)	65 (65)	
Intermediate type	66 (66)	16 (16)	
Evening type	24 (24)	19 (19)	

Values are presented as number (%) or median (range)

IBS and functional constipation were defined according to Rome IV criteria

Constipation-predominant stool pattern was defined as Bristol Stool Form Scale types 1–2

*ASM,* antiseizure medication; *FGID,* functional gastrointestinal disorder

Similarly, moderate-to-severe anxiety, as assessed by the Beck Anxiety Inventory, was significantly more frequent in PWE than in controls (54% vs. 18%, *p* < 0.001). Anxiety severity was higher in females than in males in both groups. No significant associations were observed between anxiety severity and seizure type, seizure frequency, number of ASM, classification of ASM, or epilepsy subtype within the epilepsy cohort (all *p* > 0.05) (Table 1).

Chronotype distribution revealed a marked difference between PWE and HCs. An intermediate chronotype was predominant in the PWE (66%), whereas the morning chronotype was more frequent in HCs (65%) (*p* < 0.001). Chronotype distribution was not associated with sex, seizure type, epilepsy syndrome, seizure frequency, or gastrointestinal diagnoses within the epilepsy cohort.

According to the Bristol Stool Form Scale, constipation-predominant stool types (types 1–2) were significantly more common in PWE than in HCs (27% vs. 5%, *p* < 0.001). Within the epilepsy group, constipation-predominant stool patterns were associated with higher depression and anxiety (*p* = 0.034, *p* = 0.046, respectively). Stool form was not associated with chronotype, seizure type, or epilepsy classification. Constipation-predominant stool patterns were more frequent among patients receiving polytherapy than monotherapy (*p* = 0.004) and among patients with ongoing seizures compared with seizure-free patients (*p* = 0.002).

**Fig. 1:**
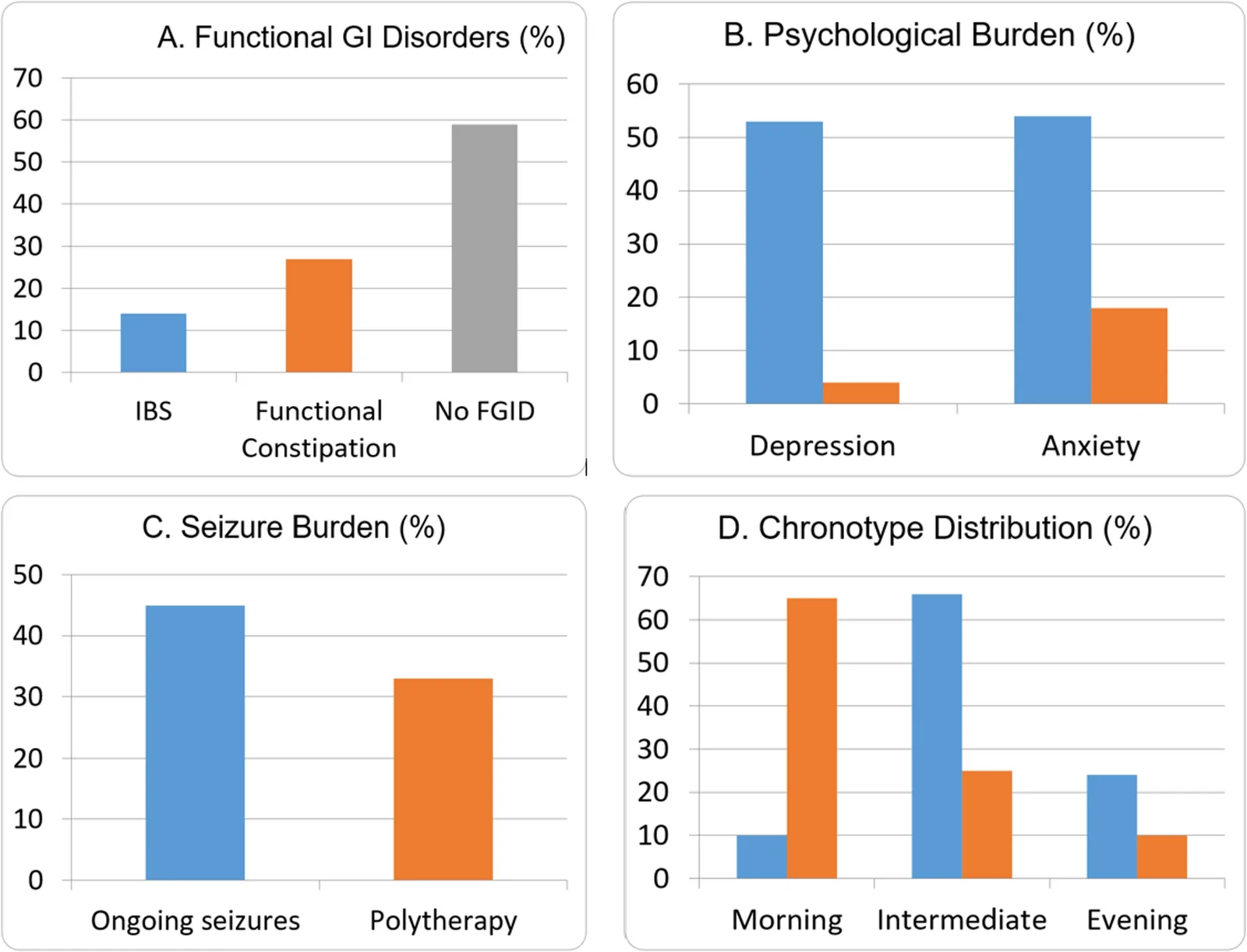
A-D: Multidimensional Burden of Epilepsy

Based on Rome IV criteria, 59 PWE (59%) had no functional gastrointestinal disorder, whereas 14 (14%) met criteria for irritable bowel syndrome (IBS) and 27 (27%) for functional constipation. In the control group, IBS and functional constipation were identified in 6% and 8% of participants, respectively. Both IBS and functional constipation were significantly more prevalent in the PWE group compared with HCs (both *p* < 0.001). None of the participants had previously sought medical care for gastrointestinal symptoms. Within the PWE group, the presence of IBS or functional constipation was associated with higher rates of depression and anxiety (*p* = 0.024 and *p* = 0.037, respectively). No significant associations were observed between Rome IV gastrointestinal diagnoses and chronotype, seizure type, epilepsy syndrome, EEG findings, MRI findings, the number of ASM, or classification of ASM class (all *p* > 0.05). Among PWE, IBS-positive and IBS-negative individuals did not differ significantly with respect to sex, seizure type, epilepsy syndrome, EEG pathology, treatment resistance, seizure control status, or chronotype (all *p* > 0.05). Among PWE, 14 (14%) met criteria for IBS, 27 (27%) for functional constipation, and 59 (59%) had no functional gastrointestinal disorder. (Fig. 1A-D)

Comparisons across these three groups demonstrated no statistically significant differences in sex distribution (*p* = 0.744), seizure type (*p* = 0.507), epilepsy syndrome classification (*p* = 0.61), seizure control status (*p* = 0.076), or chronotype distribution (*p* = 0.994) (Table 2). EEG findings differed across gastrointestinal groups. However, pathological EEG findings were observed more frequently in patients with functional constipation compared with other gastrointestinal subgroups (*p* = 0.025); this finding should be interpreted cautiously given the small subgroup sizes. No other epilepsy-related clinical characteristics showed statistically significant variation according to Rome IV gastrointestinal status. Overall, PWE demonstrated a significantly higher prevalence of functional gastrointestinal disorders, constipation-predominant stool patterns, psychological symptoms, and altered chronotype distribution compared with HCs. Within the epilepsy cohort, gastrointestinal findings were not associated with epilepsy subtype, EEG or imaging abnormalities, or treatment resistance, but were accompanied by higher psychological symptom burden.

**Table 2:** Comparison of epilepsy patients according to Rome IV gastrointestinal status

Variable	IBS (*n* = 14)	FC (*n* = 27)	No FGID (*n* = 59)	*p* value
Sex, n (%)				0.744
Female	8 (57.1)	16 (59.3)	30 (50.8)	
Male	6 (42.9)	11 (40.7)	29 (49.2)	
Seizure type, n (%)				0.507
Focal	6 (42.9)	13 (48.1)	26 (44.1)	
Generalized	6 (42.9)	5 (18.5)	17 (28.8)	
Unclassified	2 (14.3)	9 (33.3)	16 (27.1)	
Epilepsy syndrome, n (%)				0.610
Focal epilepsy	7 (50.0)	17 (63.0)	31 (52.5)	
Generalized epilepsy	7 (50.0)	4 (14.8)	20 (33.9)	
Unclassified	0 (0.0)	6 (22.2)	8 (13.6)	
EEG findings, n (%)				**0.025**
Normal	5 (35.7)	9 (33.3)	17 (28.8)	
Pathological	9 (64.3)	18 (66.7)	42 (71.2)	
Treatment resistance, n (%)				0.61
No	11 (78.6)	17 (63.0)	52 (88.1)	
Yes	3 (21.4)	10 (37.0)	7 (11.9)	
Seizure control status, n (%)				0.076
Controlled	8 (57.1)	16 (59.3)	21 (35.6)	
Uncontrolled	6 (42.9)	11 (40.7)	38 (64.4)	
Chronotype, n (%)				0.994
Morning / morning-leaning	1 (7.1)	3 (11.1)	6 (10.2)	
Intermediate	10 (71.4)	18 (66.7)	38 (64.4)	
Evening / evening-leaning	3 (21.4)	6 (22.2)	15 (25.4)	

Values are presented as number (%). IBS and functional constipation were defined according to Rome IV criteria. *FGID,* functional gastrointestinal disorder; *EEG,* electroencephalography

## Discussion

In this cross-sectional case–control study, we demonstrated that functional gastrointestinal disorders (FGIDs), particularly irritable bowel syndrome (IBS) and constipation-predominant bowel patterns, were significantly more prevalent in adults with epilepsy compared with age- and sex-matched healthy controls. These gastrointestinal disturbances co-occurred with increased psychological burden, including higher levels of anxiety and depression, and were associated with markers of greater epilepsy severity such as higher seizure frequency and polytherapy. In addition, chronotype distribution differed between groups, with patients with epilepsy more frequently exhibiting an intermediate chronotype.

The increased prevalence of FGIDs observed in our epilepsy cohort is consistent with a growing body of literature suggesting that gastrointestinal symptoms are under-recognized but common comorbidities in epilepsy. In a cohort of 147 adults with focal epilepsy, Avorio et al. reported a significantly higher frequency of FGIDs, particularly constipation and IBS, compared with controls, and demonstrated associations with seizure recurrence and impaired quality of life [4]. While the proportion of IBS in our cohort was somewhat lower, the direction and pattern of association were concordant, supporting the reproducibility of this finding across different populations.

Similarly, Lefter reviewed available evidence and emphasized that both adult and pediatric epilepsy populations show higher rates of IBS and functional constipation compared with the general population, suggesting shared pathophysiological mechanisms rather than coincidental comorbidity [19]. In pediatric epilepsy, Aydemir et al. screened 102 children and identified at least one FGID in nearly one-third of patients, with IBS being the most frequent diagnosis, further supporting the notion that gastrointestinal involvement spans across age groups [20].

Beyond prevalence, the relationship between IBS and epilepsy may be bidirectional. In a large population-based cohort study including more than 8,000 individuals with IBS, Chen et al. demonstrated an increased long-term risk of developing epilepsy compared with matched controls, suggesting that chronic gut–brain dysregulation may predispose to epileptogenesis [5]. Although causality cannot be inferred from the cross-sectional design of the present study, our findings provide clinical support for this epidemiological association.

Constipation-predominant stool patterns were significantly more frequent in patients with epilepsy in our study and were particularly prevalent among individuals with ongoing seizures and those receiving polytherapy. This observation aligns with previous reports suggesting that constipation may reflect autonomic dysfunction, reduced physical activity, cumulative medication effects, or stress-related alterations in gastrointestinal motility in epilepsy [4, 19]. Avorio et al. similarly observed that bowel movement abnormalities were associated with seizure clustering, supporting the hypothesis that gastrointestinal dysmotility may be linked to seizure activity [4]. While we did not find a significant association between constipation and specific antiseizure medication classes, constipation was more common in patients receiving multiple antiseizure drugs, suggesting that overall disease severity and treatment burden may play a more prominent role than individual drug effects. In general, gastrointestinal diagnoses were not consistently associated with epilepsy subtypes or imaging findings; however, pathological EEG findings were more frequently observed in patients with functional constipation; this finding should be interpreted with caution given the subgroup sizes.

Consistent with extensive literature, patients with epilepsy in our cohort exhibited significantly higher anxiety and depression scores compared with healthy controls. Berg et al. reported that approximately one-third of adults with epilepsy experience clinically relevant psychiatric comorbidity [1], and Tellez-Zenteno et al. demonstrated in a large population-based study that depression is independently associated with seizure frequency [2]. Importantly, we observed that IBS and functional constipation were associated with higher levels of anxiety and depression. Similar associations have been well documented in non-epilepsy populations. In a large cohort of patients with functional bowel disorders, Wu et al. showed that anxiety and depression were among the strongest predictors of IBS symptom severity [3]. Our findings extend these observations to epilepsy, supporting the concept that psychological distress and gastrointestinal symptoms may interact through shared gut–brain pathways.

Chronotype distribution also showed significant differences between epilepsy patients and the control group, with the intermediate chronotype being predominant in the epilepsy group. Although data on chronotype in adult epilepsy populations are limited, previous survey-based studies have shown that chronotype distribution and subjective sleep parameters differ in epilepsy patients compared to healthy control groups, suggesting that circadian preference may be intrinsically related to epilepsy rather than coincidental [21]. Circadian disorders play a role in seizure timing, sleep quality, and mood regulation, and changes in sleep architecture have been associated with increased seizure frequency in epilepsy [7]. Furthermore, the broader chronobiology literature has consistently associated mid- and evening chronotypes with higher anxiety and depressive symptoms [8]. Although we did not observe a direct relationship between chronotype and IBS in the epilepsy cohort, the co-occurrence of altered chronotype, psychological distress, and gastrointestinal symptoms suggests overlapping regulatory mechanisms.

Taken together, our findings highlight a clinically relevant cluster of comorbidities in adults with epilepsy involving gastrointestinal dysfunction, psychological distress, and altered chronotype. Notably, none of the patients meeting Rome IV criteria for IBS or functional constipation had previously sought medical attention for gastrointestinal symptoms, highlighting a substantial “silent burden.” This finding underscores the need for routine and proactive screening of gastrointestinal symptoms in epilepsy care. Routine screening using simple, validated questionnaires addressing gastrointestinal symptoms, mood, and chronotype may therefore facilitate earlier identification of comorbid conditions and support a more holistic, multidisciplinary management approach.

Several limitations should be acknowledged. The cross-sectional design precludes causal inference regarding the temporal relationships between epilepsy, gastrointestinal symptoms, psychological distress, and chronotype. Objective measures of circadian rhythm or sleep, such as actigraphy or polysomnography, were not included, limiting interpretation beyond self-reported chronotype. In addition, although validated instruments were used, reliance on self-report measures may introduce reporting bias. Larger longitudinal studies incorporating biological markers, including microbiome profiling and autonomic assessments, are warranted to further elucidate underlying mechanisms.

## Conclusion

In conclusion, adults with epilepsy exhibit a higher prevalence of functional gastrointestinal disorders, particularly IBS and constipation-predominant bowel patterns, alongside increased psychological burden and altered chronotype distribution. These features appear to be associated with markers of greater epilepsy severity, including higher seizure frequency and polytherapy. Our findings support an integrated clinical approach to epilepsy that extends beyond seizure control to include systematic evaluation of gastrointestinal, psychological, and chronobiological factors.

## References

[1] Berg AT, Altalib HH, Devinsky O (2017) Psychiatric and behavioral comorbidities in epilepsy: A critical reappraisal. Epilepsia 58(7):1123–1130. 10.1111/epi.13766 PMC5498258 28464309

[2] Tellez-Zenteno JF, Patten SB, Jetté N, Williams J, Wiebe S (2007) Psychiatric comorbidity in epilepsy: a population-based analysis. Epilepsia 48(12):2336–2344. 10.1111/j.1528-1167.2007.01222.x 17662062

[3] Wu JC (2012) Psychological Co-morbidity in Functional Gastrointestinal Disorders: Epidemiology, Mechanisms and Management. J Neurogastroenterol Motil 18(1):13–18. 10.5056/jnm.2012.18.1.13 PMC3271249 22323984

[4] Avorio F, Cerulli Irelli E, Morano A, Fanella M, Orlando B, Albini M, Basili LM, Ruffolo G, Fattouch J, Manfredi M, Russo E, Striano P, Carabotti M, Giallonardo AT, Severi C, Di Bonaventura C (2021) Functional Gastrointestinal Disorders in Patients With Epilepsy: Reciprocal Influence and Impact on Seizure Occurrence. Front Neurol 12:705126. 10.3389/fneur.2021.705126 PMC8377227 34421803

[5] Chen CH, Lin CL, Kao CH (2015) Irritable Bowel Syndrome Increases the Risk of Epilepsy: A Population-Based Study. Med (Baltim) 94(36):e1497. 10.1097/MD.0000000000001497 PMC4616652 26356716

[6] Ding M, Lang Y, Shu H, Shao J, Cui L (2021) Microbiota-Gut-Brain Axis and Epilepsy: A Review on Mechanisms and Potential Therapeutics. Front Immunol 12:742449. 10.3389/fimmu.2021.742449 PMC8542678 34707612

[7] Malow BA (2005) Sleep and epilepsy. Neurol Clin 23(4):1127-47. 10.1016/j.ncl.2005.07.002 16243619

[8] Adan A, Archer SN, Hidalgo MP, Di Milia L, Natale V, Randler C (2012) Circadian typology: a comprehensive review. Chronobiol Int 29(9):1153–1175. 10.3109/07420528.2012.719971 23004349

[9] Konturek PC, Brzozowski T, Konturek SJ (2011) Gut clock: implication of circadian rhythms in the gastrointestinal tract. J Physiol Pharmacol 62(2):139–150 21673361

[10] Zaccara G, Perucca E (2014) Interactions between antiepileptic drugs, and between antiepileptic drugs and other drugs. Epileptic Disord 16(4):409–431. 10.1684/epd.2014.0714 25515681

[11] Drossman DA, Hasler WL, Rome IV-Functional GI, Disorders (2016) Disorders of Gut-Brain Interaction. Gastroenterology 150(6):1257–1261. 10.1053/j.gastro.2016.03.035 27147121

[12] Lewis SJ, Heaton KW (1997) Stool form scale as a useful guide to intestinal transit time. Scand J Gastroenterol 32(9):920–924. 10.3109/00365529709011203 9299672

[13] Beck At W, Ch, Mendelson M, Mock J, Erbaugh J (1961) An inventory for measuring depression. Arch Gen Psychiatry 4:561–571. 10.1001/archpsyc.1961.01710120031004 13688369

[14] Hisli N (1989) Beck Depresyon Envanteri’nin geçerliliği ve güvenilirliği. Psikoloji Dergisi 7:3–13

[15] Beck AT, Epstein N, Brown G, Steer RA (1988) An inventory for measuring clinical anxiety: psychometric properties. J Consult Clin Psychol 56(6):893–897. 10.1037/0022-006x.56.6.893 3204199

[16] Ulusoy M, Şahin NH, Erkmen H (1998) Turkish version of the Beck Anxiety Inventory. J Cogn Psychother 12:163–172

[17] Horne JA, Ostberg O (1976) A self-assessment questionnaire to determine morningness-eveningness in human circadian rhythms. Int J Chronobiol 4(2):97–110 1027738

[18] Pündük Z, Gür H, Ercan İ (2014) Turkish MEQ validity study. Turk Psikiyatri Derg 25(2):1–9 15793697

[19] Lefter R, Ciobica A, Balmus IM (2021) Gastrointestinal functional impairments and epilepsy: Searching the possible connection mechanisms. Eur Psychiatry 64(Suppl 1):S411. 10.1192/j.eurpsy.2021.1098

[20] Aydemir Y, Carman KB, Yarar C (2020) Screening for functional gastrointestinal disorders in children with epilepsy. Epilepsy Behav 111:107267. 10.1016/j.yebeh.2020.107267 32629413

[21] Hofstra WA, Gordijn MC, van Hemert-van der Poel JC, van der Palen J, De Weerd AW (2010) Chronotypes and subjective sleep parameters in epilepsy patients: a large questionnaire study. Chronobiol Int 27(6):1271–1286. 10.3109/07420528.2010.497234 20653454

